# Directed acyclic graph kernels for structural RNA analysis

**DOI:** 10.1186/1471-2105-9-318

**Published:** 2008-07-22

**Authors:** Kengo Sato, Toutai Mituyama, Kiyoshi Asai, Yasubumi Sakakibara

**Affiliations:** 1 Japan Biological Informatics Consortium (JBIC), 2-45 Aomi, Koto-ku, Tokyo 135-8073, Japan; 2 Computational Biology Research Center (CBRC), National Institute of Advanced Industrial Science and Technology (AIST), 2-42 Aomi, Koto-ku, Tokyo 135-0064, Japan; 3 Department of Biosciences and Informatics, Keio University, 3-14-1 Hiyoshi, Kohoku-ku, Yokohama, Kanagawa 223-8522, Japan; 4 Department of Computational Biology, Graduate School of Frontier Sciences, The University of Tokyo, 5-1-5 Kashiwanoha, Kashiwa, Chiba 277-8561, Japan

## Abstract

**Background:**

Recent discoveries of a large variety of important roles for non-coding RNAs (ncRNAs) have been reported by numerous researchers. In order to analyze ncRNAs by kernel methods including support vector machines, we propose stem kernels as an extension of string kernels for measuring the similarities between two RNA sequences from the viewpoint of secondary structures. However, applying stem kernels directly to large data sets of ncRNAs is impractical due to their computational complexity.

**Results:**

We have developed a new technique based on directed acyclic graphs (DAGs) derived from base-pairing probability matrices of RNA sequences that significantly increases the computation speed of stem kernels. Furthermore, we propose profile-profile stem kernels for multiple alignments of RNA sequences which utilize base-pairing probability matrices for multiple alignments instead of those for individual sequences. Our kernels outperformed the existing methods with respect to the detection of known ncRNAs and kernel hierarchical clustering.

**Conclusion:**

Stem kernels can be utilized as a reliable similarity measure of structural RNAs, and can be used in various kernel-based applications.

## Background

Recent discoveries of a large variety of important roles for non-coding RNAs (ncRNAs), including gene regulation or maturation of mRNAs, rRNAs and tRNAs, have been reported by many researchers. Most functional ncRNAs form secondary structures related to their functions, and secondary structures without pseudoknots can be modeled by stochastic context-free grammars (SCFGs) [1,2]. Therefore, several computational methods based on SCFGs have been developed for modeling and analyzing functional ncRNA sequences [3-14]. These grammatical methods work very well if the secondary structures of the target ncRNAs are modeled successfully. However, it is difficult to build such stochastic models since it is necessary to construct complicated models, to prepare the number of training sequences, and/or to obtain prior knowledge for some families containing non-uniform and/or non-homologous sequences such as snoRNA families. Thus, we need more robust methods for performing structural ncRNA analysis. On the other hand, support vector machines (SVMs) and other kernel methods are being actively studied, and have been proposed for solving various problems in many research fields, including bioinformatics [15]. These methods are more robust than other existing methods, and we therefore considered using kernel methods including SVMs instead of the grammatical methods to analyze functional ncRNAs.

Several kernels for ncRNA sequences have been developed so far [16-19]. Kin *et al*. have proposed marginalized count kernels for RNA sequences [16]. Their kernels calculate marginalized count vectors of base-pair features under SCFGs trained with a given dataset, and compute the inner products. Therefore, marginalized count kernels inherit the drawback of the grammatical methods. Washietl *et al*. have developed a program called RNAz, which detects structurally conserved regions from multiple alignments by using SVMs [17]. RNAz employs the averaged *z*-score of the minimum free energy (MFE) for each sequence and structure conservation index (SCI). Assuming that MFE for the common secondary structure is close to that for each sequence if a given multiple alignment is structurally conserved, SCI is defined as the rate of MFE for the common secondary structure to the averaged MFE for each sequence. These features allow for the detection of structurally conserved regions. However, since these features cannot measure the structural similarities between RNA sequences, it is difficult to apply them to other aspects of structural RNA analysis, such as detecting particular families. Several works which involve some helpful features specific to given target families (e.g. miRNAs and snoRNAs) have been proposed [18,19]. These family-specific methods perform well in detecting their target families. However, in order to apply this strategy to other families, it is necessary to develop new features for every family.

For the purpose of analyzing ncRNAs using kernel methods including support vector machines, we have proposed *stem kernels*, which extend the string kernels to measure the similarities between two RNA sequences from the viewpoint of secondary structures [20]. The feature space of the stem kernels is defined by enumerating all possible common base pairs and stem structures of arbitrary lengths. However, since the computational time and memory size required for the naive implementation of stem kernels are of the order of *O*(*n*^4^), where *n *is the length of the inputted RNA sequence, applying stem kernels directly to large data sets of ncRNAs is impractical.

Therefore, we develop a new technique based on directed acyclic graphs (DAGs) derived from base-pairing probability matrices of RNA sequences, which significantly reduces the computational time of stem kernels. The time and space complexity of this method are approximately of the order of *O*(*n*^2^). Furthermore, we propose profile-profile stem kernels for multiple alignments of RNA sequences, which utilize base-pairing probability matrices for multiple alignments instead of those for individual sequences.

## Methods

In this section, we propose new kernels for analyzing ncRNAs. First, an outline of our previous work is provided, after which the proposed new technique based on directed acyclic graphs (DAGs) derived from base-pairing probability matrices of RNA sequences is described. Finally, the proposed kernels are extended to kernels for multiple alignments of RNA sequences by utilizing averaged base-pairing probability matrices.

### Naive stem kernel algorithms

Before proposing the new method, we briefly describe stem kernels which have been proposed as an extension of the string kernels for measuring the similarities between two RNA sequences from the viewpoint of secondary structures [20]. The feature space of the stem kernels is defined by enumerating all possible common base pairs and stem structures of arbitrary lengths. The stem kernel calculates the inner product of common stem structure counts. In other words, the more stem structures two RNA sequences have in common, the more similar they are. However, the time needed for the explicit enumeration of all substructures obviously grows exponentially, which renders this method infeasible for long sequences. We have therefore developed an algorithm for calculating stem kernels which is based on the dynamic programming technique. For an RNA sequence **x **= *x*_1_*x*_2 _... *x*_*n *_(*x*_*k *_∈ {A, C, G, U}), we denote a contiguous subsequence *x*_*j *_... *x*_*k *_by **x **[*j*, *k*], and the length of **x **by |**x**|. The empty sequence is indicated by *∊*. For a base *a*, the complementary base is denoted as $\bar{a}$. For a string **x **and a base *a*, **x***a *denotes the concatenation of **x **and *a*. For two RNA sequences **x **and **x***'*, the stem kernel *K *is defined recursively as follows:

(1) $$\begin{array}{l} \begin{array}{cc} K(\epsilon ,x^{\prime })=K(x,\epsilon )=1, & \text{for }\forall x,x^{\prime } \end{array},\\ K(xa,x^{\prime })=K(x,x^{\prime })+\sum_{x_{k}=\bar{a}}\sum_{i<j\text{ s}.\text{t}.\;{x^{\prime }}_{i}=\bar{a},{x^{\prime }}_{j}=a}K(x[k+1,|x|],x^{\prime }[i+1,j-1]). \end{array}$$

Both the time and the memory required for the calculation *K*(**x**, **x***'*) are of the order of *O*(|**x**|^2^|**x***'*|^2^), which renders this method impractical for applying to large data sets of ncRNAs.

### Stem kernels with DAG representation

**Figure 1:**
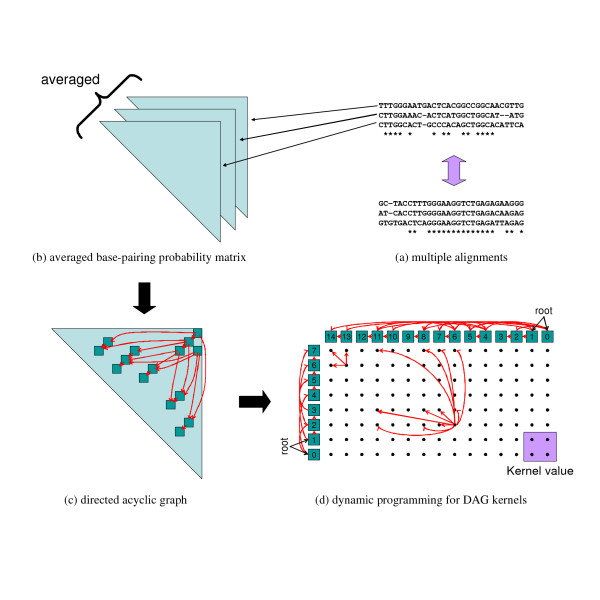
**Averaged base-paring probability matrices and DAG kernels using the dynamic programming technique enable us to calculate profile-profile stem kernels for multiple alignments of RNA sequences**. (a) Given a pair of multiple alignments, (b) Calculate the base-paring probability matrices for each sequence in the multiple alignments and average these base-pairing probabilities with respect to the columns of each alignment. (c) Build a DAG for the averaged base-pairing probability matrix, where each vertex corresponds to a base pair whose probability is above a predefined threshold. (d) Calculate a kernel value for a pair of DAGs for the multiple alignments by using the DAG kernel and the dynamic programming technique.

Here, we develop a new technique based on directed acyclic graphs (DAGs) derived from base-pairing probability matrices of RNA sequences, which significantly reduces the time needed for computing stem kernels. Figure 1 contains a diagram illustrating the calculation of the new kernels.

First, for each RNA sequence **x **= *x*_1_*x*_2 _... *x*_*n*_, we calculate a base-pairing probability matrix *P*^**x **^using the McCaskill algorithm [21]. We denote the base-pairing probability of (*x*_*i*_, *x*_*j*_) by $P_{ij}^{x}$, which is defined as:

(2) $$P_{ij}^{x}=E[I_{ij}|x]=\sum_{y\in Y(x)}p(y|x)I_{ij}(y),$$

where Y(**x**) is an ensemble of all possible secondary structures of **x**, *p*(*y*|**x**) is the posterior probability of *y *given **x**, and *I*_*ij*_(*y*) is an indicator function, which equals 1 if the *i*-th and the *j*-th nucleotides form a base-pair in *y *or 0 otherwise. We employ the Vienna RNA package [22] for computing these expected counts (2) using the McCaskill algorithm.

Subsequently, we build a DAG for the base-pairing probability matrix, where each vertex corresponds to a base pair whose probability is above a predefined threshold p*. Let *G*_**x **_= (*V*_**x**_, *E*_**x**_) be the DAG for an RNA sequence **x**, where *V*_**x **_and *E*_**x **_are vertices and edges in the DAG *G*_**x**_, respectively. For each *v*_*i *_= (*k*, *l*) ∈ *V*_**x**_, (*x*_*k*_, *x*_*l*_) is a likely base pair, in other words, $P_{kl}^{x}\geq p^{*}$. Each *e*_*ij *_∈ *E*_**x **_is an edge from vertex *v*_*i *_to vertex *v*_*j*_.

For vertices *v*_*i *_= (*k*, *l*) and *v*_*i' *_= (*k'*, *l'*), we can define a partial order, *v*_*i *_≺ *v*_*i' *_if and only if *k *<*k' *and *l *> *l'*. An edge *e*_*ii' *_connects vertices *v*_*i *_and *v*_*i' *_if and only if *v*_*i *_≺ *v*_*i' *_and there exists no *v*_*j *_∈ *V*_**x **_such that *v*_*i *_≺ *v*_*j *_≺ *v*_*i'*_.

Finally, we calculate a kernel value between two DAGs representing RNA structure information through the DAG kernel using a dynamic programming technique. The vertices in the DAG can be numbered in a topological order such that for every edge *e*_*ij*_, *i *<*j *is satisfied, in other words, there are no directed paths from *v*_*j *_to *v*_*i *_if *i *<*j*. Thus, we can apply the dynamic programming technique as follows:

(3) $$\begin{array}{l} K(G_{x},G_{x^{\prime }})=\sum_{v_{i}\in root(G_{x}),v_{i^{\prime }}\in root(G_{x^{\prime }})}r(i,i^{\prime })\\ r(i,i^{\prime })=\{\begin{array}{ll} K_{v}(v_{i},v_{i^{\prime }})+g_{v}(v_{i})+g_{v}(v_{i^{\prime }}) & \left(∄j,j^{\prime }\text{ s}.\text{t}.\;j>i,j^{\prime }>i^{\prime }\right)\\ K_{v}(v_{i},v_{i^{\prime }})+g_{v}(v_{i})\sum_{j>i}g_{e}(e_{ij})r(j,i^{\prime })+g_{v}(v_{i^{\prime }}) & \left(∄j^{\prime }\text{ s}.\text{t}.\;j^{\prime }>i^{\prime }\right)\\ K_{v}(v_{i},v_{i^{\prime }})+g_{v}(v_{i})+g_{v}(v_{i^{\prime }})\sum_{j^{\prime }>i^{\prime }}g_{e}(e_{i^{\prime },j^{\prime }})r(i,j^{\prime }) & \left(∄j\text{ s}.\text{t}.\;j>i\right)\\ K_{v}(v_{i},v_{i^{\prime }})\sum_{j>i,j^{\prime }>i^{\prime }}K_{e}(e_{ij},e_{i^{\prime }j^{\prime }})r(j,j^{\prime }) & \\ +g_{v}(v_{i})\sum_{j>i}g_{e}(e_{ij})r(j,i^{\prime })+g_{v}(v_{i^{\prime }})\sum_{j^{\prime }>i^{\prime }}g_{e}(e_{i^{\prime }j^{\prime }})r(i,j^{\prime }) & \left(\text{otherwise}\right) \end{array} \end{array}$$

where *root*(*G*) is a set of vertices which have no incoming edges, *K*_*v *_and *K*_*e *_are kernel functions for vertices and edges, respectively, and *g*_*v *_and *g*_*e *_are gap penalties for vertices and edges, respectively. *K *calculates the sum of kernel values for all pairs of possible substructures of *G*_**x **_and *G*_**x***'*_. Each of these kernel values is composed of the product of the subkernels *K*_*v*_, *K*_*e*_, *g*_*v *_and *g*_*e*_. Therefore, *K *is a convolution kernel and is positive semi-definite if *K*_*v *_and *K*_*e *_are also positive semi-definite [23].

The time and the memory required for the computation of *K *are of the order of *O*(*c*^2^|*V*_**x**_||*V*_**x***'*_|) and *O*(|*V*_**x**_||*V*_**x***'*_|), respectively, where *c *is the maximum out-degree of *G*_**x **_and *G*_**x***'*_. We can control |*V*_**x**_| using the predefined threshold for base pairs, p*. When p* = 0, *V*_**x **_contains all possible base pairs, i.e., |*V*_**x**_| = *n*(*n *- 1)/2. When p* > 0, since each base can take part in *V*_**x **_at most 1/p* times, |*V*_**x**_| is proportional to *n *of the length of the RNA sequence **x**. Since in many cases *c *≪ |*V*_**x**_|, the time and the memory required for this algorithm are approximately of the order of *O*(*n*^2^) for sufficiently large values of p*.

Several choices of sub-kernels *K*_*v*_, *K*_*e*_, *g*_*v *_and *g*_*e *_in Eq. (3) are available. In order to connect the DAG-based stem kernels to the naive stem kernels calculated from Eq. (1), we first define simple sub-kernels as follows:

(4) $$K_{v}(v,v^{\prime })=\{\begin{array}{ll} 1 & \left(\begin{array}{l} {\bar{x}}_{k}=x_{l}\text{ and }(x_{k},x_{l})=({x^{\prime }}_{k^{\prime }},{x^{\prime }}_{l^{\prime }})\\ \text{for }v=(k,l)\in V_{x}\text{ and }v^{\prime }=(k^{\prime },l^{\prime })\in V_{x^{\prime }} \end{array}\right)\\ 0 & \left(\text{otherwise}\right) \end{array}$$

(5) $$K_{e}(e,e^{\prime })=\{\begin{array}{ll} 1 & \left(e\in E_{x}\text{ and }e^{\prime }\in E_{x^{\prime }}\right)\\ 0 & \left(\text{otherwise}\right) \end{array}$$

(6) *g*_*v*_(*v*) = 1,   ∀*v *∈ *V*_**x **_∪ *V*_**x***'*_

(7) *g*_*e*_(*e*) = 1,   ∀*e *∈ *E*_**x **_∪ *E*_**x***'*_.

When p* → 0, the DAG-based stem kernels calculated form Eq. (3) with the above sub-kernels approach the naive stem kernels calculated from Eq. (1) since both Eqs. (1) and (3) designate recursive traversal to all substructures of **x **and **x***' *in the sense of the partial order ≺, and when p* = 0, the substructures of **x **and **x***' *for both kernels which contribute kernel values are identical to each other due to these sub-kernels. More sophisticated kernels can be constructed using substitution scoring matrices, as well as local alignment kernels [24]:

(8) $$\begin{array}{l} K_{v}(v,v^{\prime })=\exp\left(P_{kl}^{x}P_{k^{\prime }l^{\prime }}^{x^{\prime }}\cdot \alpha \cdot S(x_{k},x_{l},{x^{\prime }}_{k^{\prime }},{x^{\prime }}_{l^{\prime }})\right)\\ \;(\text{for }v=(k,l)\in V_{x}\text{ and }v^{\prime }=(k^{\prime },l^{\prime })\in V_{x^{\prime }}), \end{array}$$

(9) $$K_{e}(e,e^{\prime })=\{\begin{array}{ll} \gamma^{n(e)+n(e^{\prime })} & \left(e\in E_{x}\text{ and }e^{\prime }\in E_{x^{\prime }}\right)\\ 0 & \left(\text{otherwise}\right) \end{array}$$

(10) *g*_*v*_(*v*) = *γ*^2^,   ∀*v *∈ *V*_**x **_∪ *V*_**x***'*_

(11) *g*_*e*_(*e*) = *γ*^*n*(*e*)^,   ∀*e *∈ *E*_**x **_∪ *E*_**x***'*_,

where $S(x_{l},x_{k},{x^{\prime }}_{k^{\prime }},{x^{\prime }}_{l^{\prime }})$ is a substitution scoring function from a base pair (*x*_*l*_, *x*_*k*_) to a base pair $\left({x^{\prime }}_{k^{\prime }},{x^{\prime }}_{l^{\prime }}\right)$, *α *> 0 is a weight parameter for base pairs, *γ *> 0 is the decoy factor for loop regions, and *n*(*e*) is the number of nucleotides in the loop region enclosed by base pairs at both ends of an edge *e*.

In our experiments, we employed the RIBOSUM 80-65 [9] for *S*, and p* = 0.01, *α *= 0.1, *γ *= 0.4, which were optimized by cross-validation tests. In order to prevent sequence length bias, we normalize our kernels *K *as follows:

$$K^{\prime }(G_{x},G_{x^{\prime }})=\frac{K(G_{x},G_{x^{\prime }})}{\sqrt{K(G_{x},G_{x})K(G_{x^{\prime }},G_{x^{\prime }})}}.$$

Stem kernels can be applied only to RNA secondary structures. However, primary sequences are still important for calculating the similarities between a pair of RNA sequences. Therefore, in order to take into account both primary sequences and secondary structures, we combine our stem kernels with the local alignment kernels by adding them.

### Profile-profile stem kernels

If multiple alignments of homologous RNA sequences are available, we can calculate their base-paring probability matrices more precisely by taking the averaged sum of individual base-pairing probability matrices in accordance with the given multiple alignment [25]. The algorithm of the DAG-based stem kernels for a pair of RNA sequences can be extended to that for a pair of multiple alignments of RNA sequences using averaged base-pairing probability matrices. Since the method of the averaged base-paring probability matrices has been proven to be accurate and robust by Kiryu *et al*. [25], we can expect this method to improve the proposed stem kernel method. We call these profile-profile stem kernels.

We denote the *i*-th column of a multiple alignment **A **by **A**_*i*_, a nucleotide in **A**_*i *_of the *j*-th sequence by *a*_*ij*_, and the number of aligned sequences in **A **by *num*(**A**). We can calculate the averaged base-pairing probability matrix of a given multiple alignment **A **as follows:

$$\begin{array}{l} P_{kl}^{A}=\frac{1}{num(A)}\sum_{x\in A}{P^{\prime }}_{kl}^{x},\\ {P^{\prime }}_{kl}^{x}=\{\begin{array}{ll} P_{\rho (k)\rho (l)}^{x^{\prime }} & \left(\text{for either of }x_{k}\text{ and }x_{l}\text{ are not gaps}\right)\\ 0 & (\text{otherwise}), \end{array} \end{array}$$

where **x***' *is the sequence **x **with all gaps removed and *ρ*(*k*) is an index on **x***' *of the *k*-th column of **A**. After constructing $P_{kl}^{A}$, we can build DAGs, and the kernel *K*_*v *_for columns can be calculated by replacing the substitution function *S *in Eq. (9) with

$$\begin{array}{l} S(A_{k},A_{l},{A^{\prime }}_{k^{\prime }},{A^{\prime }}_{l^{\prime }})=\frac{1}{num(A)num(A^{\prime })}\sum_{i=1}^{num(A)}\sum_{i^{\prime }=1}^{num(A^{\prime })}S^{\prime }(a_{ki},a_{li},{a^{\prime }}_{k^{\prime }i^{\prime }},{a^{\prime }}_{l^{\prime }i^{\prime }})\\ S^{\prime }(a_{ki},a_{li},{a^{\prime }}_{k^{\prime }i^{\prime }},{a^{\prime }}_{l^{\prime }i^{\prime }})=\{\begin{array}{ll} S(a_{ki},a_{li},{a^{\prime }}_{k^{\prime }i^{\prime }},{a^{\prime }}_{l^{\prime }i^{\prime }}) & \left(\text{any of }a_{ki},a_{li},{a^{\prime }}_{k^{\prime }i^{\prime }},\text{ and }{a^{\prime }}_{l^{\prime }i^{\prime }}\text{ are not gaps}\right)\\ 0 & (\text{otherewise}). \end{array} \end{array}$$

## Results and Discussion

In this section, we present some of the results of our experiments in order to confirm the validity of our method as well as a discussion of those results.

### Discrimination with SVMs and other kernel machines

We performed several experiments in which SVMs based on our kernel attempted to detect known ncRNA families. The accuracy was assessed using the specificity (*SP*) and the sensitivity (*SN*), which are defined as follows:

$$\begin{array}{cc} SP=\frac{TN}{TN+FP}, & SN=\frac{TP}{TP+FN}, \end{array}$$

where *TP *is the number of correctly predicted positives, *FP *is the number of incorrectly predicted positives, *TN *is the number of correctly predicted negatives, and *FN *is the number of incorrectly predicted negatives. Furthermore, the area under the receiver operating characteristic (ROC) curve, i.e., the ROC score, was also used for evaluation. The ROC curve plots the true positive rates (= *SN*) as a function of the false positive rates (= 1 - *SP*) for varying decision thresholds of a classifier.

**Figure 2:**
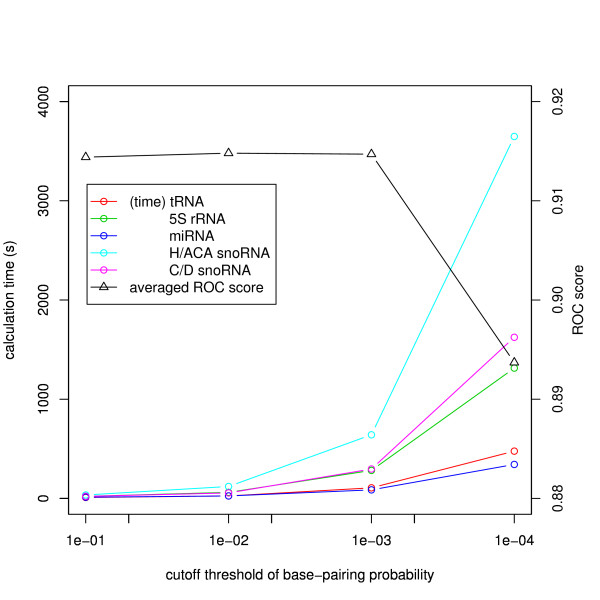
**Calculation time and ROC scores for various cutoff threshold values of the base-pairing probabilities**. We timed the DAG-based stem kernels in calculating a kernel matrix for each family of the training set containing 100 positives and 100 negatives, and confirmed the accuracy of their discrimination through the ROC scores.

In our first experiment, the discrimination ability and the execution time of the stem kernels were tested on our previous dataset used in [20], which includes five RNA families: tRNAs, miRNAs (precursor), 5S rRNAs, H/ACA snoRNAs, and C/D snoRNAs. We chose 100 sequences in each RNA family from the Rfam database [26] as positive samples such that the pairwise identity was not above 80% for any pair of sequences, and 100 randomly shuffled sequences with the same dinucleotide composition as the positives were generated as negative samples for each family. The discrimination performance was evaluated using 10-fold cross validation. In order to determine an appropriate cutoff threshold for the base-pairing probabilities p*, we performed the experiments for various values of p* ∈ {0.1, 0.01, 0.001, 0.0001}. Figure 2 shows the accuracy and the calculation time for each threshold. Since the accuracy for p* = 0.01 was slightly better than that for the other values, and the calculation time in this case was acceptable for practical use, we fixed p* = 0.01 as the default cutoff threshold of the base-pairing probabilities. Then, we compared the DAG-based stem kernels with the naive stem kernels. The experimental results shown in Table 1 indicate that the DAG-based kernels are significantly faster than the naive kernels owing to the approximation by a predefined threshold of the base-pairing probability. Furthermore, in spite of using an approximation, the DAG-based kernels are slightly more accurate than the naive kernels due to the convolution with the local alignment kernels and the removal of low-likelihood base pairs which may create noise.

**Table 1:** Comparison of the discrimination capabilities of the naive stem kernels and the DAG-based stem kernels.

	Naive stem kernels				DAG-based stem kernels			
		
ncRNA type	ROC	SP	SN	Time (s)	ROC	SP	SN	Time (s)
tRNA	0.97	0.82	0.94	0.9	0.98	0.93	0.86	9.9 × 10^-4^
5S rRNA	0.97	0.97	0.74	5.1	1.00	1.00	0.95	2.2 × 10^-3^
miRNA	0.88	0.65	0.88	1.6	0.86	0.88	0.69	9.7 × 10^-4^
H/ACA snoRNA	0.80	0.80	0.54	12.8	0.89	0.90	0.72	4.1 × 10^-3^
C/D snoRNA	0.78	0.55	0.79	4.7	0.87	0.91	0.71	2.0 × 10^-3^

The dataset contains five RNA families: tRNAs, miRNAs, 5S rRNAs, H/ACA snoRNAs, and C/D snoRNAs. ncRNA type: name of the target ncRNA family. ROC: ROC score, equal to the area under the ROC curve. SP: specificity of the discrimination of the target ncRNA family. SN: sensitivity of the discrimination of the target ncRNA family. Time: averaged time for each kernel computation on a 2.0 GHz AMD Opteron processor.

Next, we performed the experiment on a large dataset including multiple alignments, which was used to train RNAz [17]. This dataset includes 12 ncRNA families of 7,169 original alignments, extracted from the Rfam database [26], with the exception of the single-recognition particle (SRP) RNA and RNAseP, which were extracted from [27,28]. Each alignment consists of two to ten sequences aligned by CLUSTAL-W [29], and the mean pairwise identities are between 50% and 100%. The dataset also includes 7,169 negatives, which were generated from the original alignments by shuffling the columns, where the conservation rate on each column was preserved [30]. In this experiment, for each RNA family, SVMs trained the model which distinguishes the original alignments of a target RNA family from all other original and shuffled alignments in the dataset. We compared the profile-profile stem kernels with the local alignment kernels [24], which only consider primary sequences of RNAs. Subsequently, we extended the local alignment kernels using the same technique as in the case of the profile-profile stem kernels in order to account for multiple alignments.

The discrimination performance of both kernels was evaluated with 10-fold cross-validation. Table 2 presents the experimental results for this dataset. The stem kernels attained nearly perfect discrimination for all families in this dataset, while the local alignment kernels failed to discriminate some families. The performance with respect to tmRNA and RNAse P in terms of sensitivity was especially low. Furthermore, the stem kernels collected a smaller number of support vectors in comparison with the local alignment kernels due to the robustness of the stem kernels with respect to secondary structures. This is a desirable feature since the prediction process of SVMs requires only support vectors for the calculation of kernel values against an input sequence.

**Table 2:** Non-coding RNA detection using SVMs in comparing the stem kernels with the local alignment kernels.

			Stem kernels				Local alignment kernels			
		
ncRNA type	Rfam Accession	N	ROC	SP	SN	nSV	ROC	SP	SN	nSV
5S ribosomal RNA	RF00001	449	1.000	1.000	0.996	164.9 (1.3)	1.000	1.000	0.996	4013.0 (31.1)
U2 spliceosomal RNA	RF00004	566	0.999	1.000	0.993	631.2 (4.9)	0.999	1.000	0.986	4117.5 (31.9)
tRNA	RF00005	495	0.998	1.000	0.998	234.8 (1.8)	1.000	1.000	0.998	4287.2 (33.2)
Hammerhead ribozyme III	RF00008	588	1.000	1.000	0.997	221.2 (1.7)	1.000	1.000	0.997	2452.1 (19.0)
U3 snoRNA	RF00012	471	1.000	1.000	0.996	266.2 (2.1)	0.998	1.000	0.870	4665.3 (36.2)
U5 spliceosomal RNA	RF00020	510	1.000	1.000	0.996	525.5 (4.1)	1.000	1.000	0.994	4060.0 (31.5)
tmRNA	RF00023	730	1.000	1.000	0.997	685.8 (5.3)	0.975	1.000	0.037	4677.7 (36.2)
Group II intron	RF00029	604	1.000	1.000	0.993	482.7 (3.7)	1.000	1.000	0.990	4217.3 (32.7)
mir-10	RF00104	620	1.000	1.000	0.998	59.5 (0.5)	1.000	1.000	0.998	159.6 (1.2)
U70 snoRNA	RF00156	608	0.999	1.000	0.990	195.0 (1.5)	0.999	1.000	0.992	3811.8 (29.5)
RNAse P	-	656	1.000	1.000	0.991	490.6 (3.8)	0.905	1.000	0.018	4729.2 (36.6)
SRP RNA	-	872	1.000	1.000	0.995	441.5 (3.4)	0.908	1.000	0.900	4373.9 (33.9)

Total		7169	1.000	1.000	0.995	4398.9 (2.9)	0.977	1.000	0.788	45564.6 (29.5)

ncRNA type: name of the target ncRNA family. Rfam Accession: accession number of the target ncRNA family in Rfam. N: number of alignments. ROC: ROC score, equal to the area under the ROC curve. SP: specificity of the discrimination of the target ncRNA family. SN: sensitivity of the discrimination of the target ncRNA family. nSV: number of support vectors collected in the training processes and their rates against the numbers of the training alignments within parentheses.

In addition, we employed another kernel machine instead of SVM, called support vector data description (SVDD) [31], which calculates a spherically shaped boundary around a dataset so as to increase the robustness against outliers without the need for negative examples. In other words, SVDD does not need to generate artificial negative examples. Many applications of SVMs to biological problems require the artificial generation of negative examples such as shuffled positive sequences. However, since most artificial negatives can be easily distinguished from positives in many cases, the generation of artificial negative examples is a crucial problem to attaining practical prediction performance [32]. In this regard, SVDD can avoid this problem by using only positive examples. We applied SVDD instead of SVMs to the above dataset. Table 3 shows the surprising discovery that there is little difference in the accuracy of SVMs and SVDD. This result indicates that negative examples produced by shuffling the alignments make a very small contribution to learning the classifiers with our kernels. Furthermore, the number of support vectors in SVDD decreased significantly in comparison to SVMs.

**Table 3:** Non-coding RNA detection using SVDD in comparing the stem kernels with the local alignment kernels.

			Stem kernels				Local alignment kernels			
		
ncRNA type	Rfam Accession	N	ROC	SP	SN	nSV	ROC	SP	SN	nSV
5S ribosomal RNA	RF00001	449	1.000	1.000	0.940	27.8 (6.9)	1.000	1.000	0.886	48.4 (12.0)
U2 spliceosomal RNA	RF00004	566	0.997	0.999	0.912	51.8 (10.2)	0.999	1.000	0.844	92.0 (18.1)
tRNA	RF00005	495	0.983	0.948	0.939	26.8 (6.0)	0.999	0.999	0.853	67.0 (15.0)
Hammerhead ribozyme III	RF00008	588	1.000	0.998	0.971	14.2 (2.7)	1.000	1.000	0.968	19.3 (3.6)
U3 snoRNA	RF00012	471	1.000	1.000	0.915	36.3 (8.6)	0.959	1.000	0.775	95.5 (22.5)
U5 spliceosomal RNA	RF00020	510	0.999	0.998	0.939	30.3 (6.6)	1.000	1.000	0.882	57.2 (12.5)
tmRNA	RF00023	730	1.000	1.000	0.881	83.1 (12.6)	0.757	1.000	0.037	636.5 (96.9)
Group II intron	RF00029	604	0.996	0.989	0.942	30.9 (5.7)	0.999	1.000	0.922	48.7 (9.0)
mir-10	RF00104	620	1.000	1.000	0.977	13.3 (2.4)	1.000	1.000	0.984	10.7 (1.9)
U70 snoRNA	RF00156	608	0.998	0.996	0.952	25.5 (4.7)	1.000	1.000	0.951	29.0 (5.3)
RNAse P	-	656	0.998	1.000	0.887	66.2 (11.2)	0.629	1.000	0.006	587.5 (99.5)
SRP RNA	-	872	1.000	1.000	0.939	54.4 (6.9)	0.994	1.000	0.881	95.3 (12.1)

Total		7169	0.998	0.995	0.932	460.6 (7.1)	0.938	1.000	0.729	1787.1 (27.7)

ncRNA type: name of the target ncRNA family. Rfam Accession: accession number of the target ncRNA family in Rfam. N: number of alignments. ROC: ROC score, equal to the area under the ROC curve. SP: specificity of the discrimination of the target ncRNA family. SN: sensitivity of the discrimination of the target ncRNA family. nSV: number of support vectors collected in the training processes and their rates against the numbers of the training alignments within parentheses.

In this section, we trained SVMs with the stem kernels to detect particular ncRNA families. On the other hand, the SVMs in RNAz are trained to detect any structural ncRNAs, including unknown ncRNAs [17]. In order to demonstrate that RNAz is capable of discovering unknown ncRNAs with no bias toward the ncRNA families of the training set, SVMs were trained by excluding particular families of ncRNAs, and were used for classifying the excluded ncRNAs and the shuffled negatives. We attempted the same training scheme as described in [17] to investigate the ability of the stem kernels to discover unknown ncRNAs using the same dataset as in the experiment of Table 2. As a result, the ROC scores in this test were 0.699 for the stem kernels, 0.582 for the local alignment kernels, and 0.949 for RNAz. This result suggests that the ability of stem kernels to discover unknown ncRNAs is weaker than that of RNAz. The key feature in discovering unknown structural ncRNAs is to detect evolutionary conserved structures in multiple sequence alignments. The SCI used in RNAz directly assesses the structure conservation in multiple alignments, and it contributes to the ability of detecting unknown structural ncRNAs. However, since the SCI cannot measure the structural similarities between RNA sequences, it is difficult to apply it to other aspects of structural RNA analysis, such as detecting particular families. On the other hand, the stem kernels evaluate common stem structures between two multiple alignments, in other words, the stem kernels are not the measure of the structure conservation, but rather are the measure of the structural similarity between ncRNAs. Therefore, the stem kernels can be applied to various kernel methods including not only SVMs but also kernel principal component analysis (KPCA), kernel canonical correlation analysis (KCCA), and so on [15].

### Remote homology search

Furthermore, we conducted a remote homology search of ncRNAs using SVMs with our kernel. Our kernel method was compared with INFERNAL [7] based on profile SCFGs. INFERNAL has been recommended for RNA homology search by the benchmark of currently available RNA homology search tools called BRAliBase III [33]. This benchmark dataset contains tRNAs, 5S rRNAs and U5 spliceosomal RNAs, which have relatively conserved sequences and/or secondary structures, whereby both INFERNAL and our kernel can easily detect homologs (data not shown).

Therefore, we performed a more practical remote homology search on the dataset shown in Table 4, which includes 47 sequences of H/ACA snoRNAs and 41 sequences of C/D snoRNAs in *C. elegans *from the literature [34]. These mean pairwise identities are too low to be discovered by existing methods. For each family, non-homologs were generated by shuffling every sequence 10 times. The shuffling processes preserved dinucleotide frequencies. Twenty query sets of 5 and 10 sequences were sampled from each family, respectively. Using these query sets, we attempted to search for homologs among all of the original and the shuffled sequences.

**Table 4:** Summary of the dataset for the experiment of the remote homology search.

ncRNA type	N	Length	%id
H/ACA snoRNA	47	145.1	29%
C/D snoRNA	41	84.6	30%

ncRNA type: name of the target ncRNA family. N: number of sequences in the dataset of the target ncRNA family. length: average length of the sequences. %id: mean pairwise identity of the dataset.

For INFERNAL, each query was aligned by CLUSTAL-W [29], folded by RNAalifold [35], and converted into a covariance model (CM). The CM searched for homologous sequences in the dataset, calculating a bit score for each sequence. A ROC curve can be plotted using the bit scores as decision values.

For the stem kernel, every sequence for each query was shuffled 10 times in order to generate negative samples. Then, the SVM with the stem kernel learned the discrimination model from the query and the negatives. The model searched for homologous sequences in the dataset, calculating an SVM class probability for each sequence. A ROC curve can be plotted in this case using SVM class probabilities as decision values.

**Figure 3:**
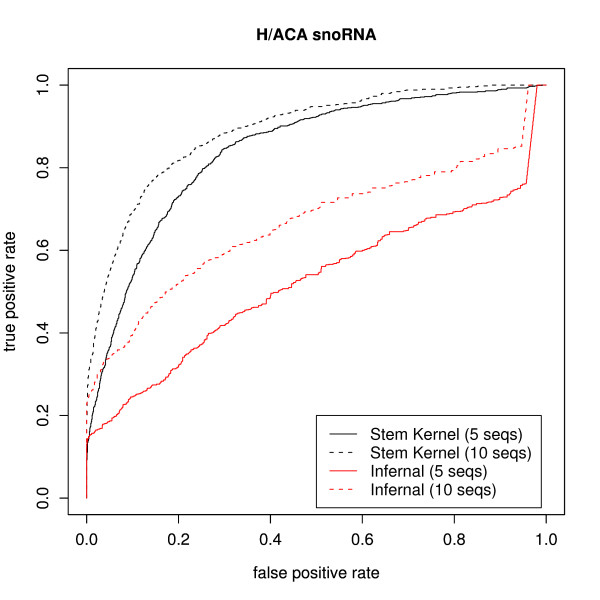
**ROC curves of the remote homology searches of H/ACA snoRNAs in *C. elegans *from **[34]**in comparing our kernels with that of INFERNAL**. For every 20 query sets of 5 (or 10) sequences, we search for homologous sequences among all of the original and the shuffled sequences.

**Figure 4:**
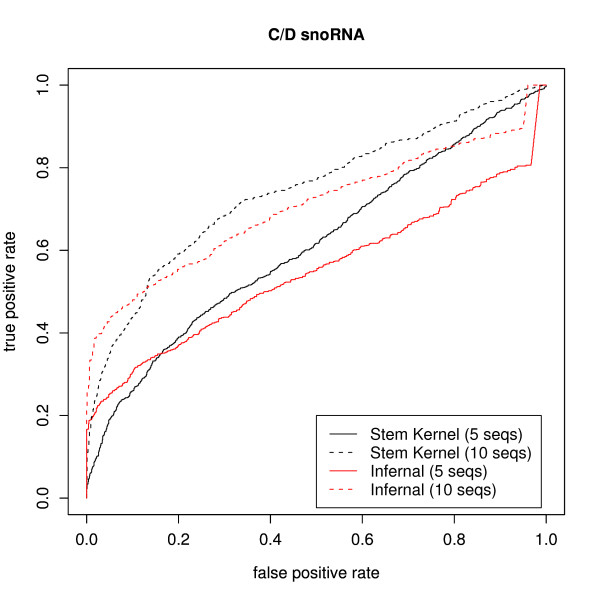
**ROC curves of the remote homology searches of C/D snoRNAs in *C. elegans *from **[34]**in comparing our kernels with that of INFERNAL**. For each of 20 query sets of 5 (or 10) sequences, we search for homologous sequences among all of the original sequences and the shuffled sequences.

Figures 3 and 4 display the ROC curves of the homology searches of H/ACA snoRNAs and C/D snoRNAs by INFERNAL and SVMs with the stem kernels. The stem kernel produced more precise results than INFERNAL with respect to searching the target families for homologs. In particular, in the H/ACA snoRNAs experiment, the stem kernel was capable of detecting them accurately even with queries of 5 sequences. However, the accurate identification of C/D snoRNAs was problematic for both methods, which can be attributed to the poor secondary structures of C/D snoRNAs. In fact, the identification of C/D snoRNAs is difficult for many structure-based methods.

Note that the sequences in the datasets shown in Table 4 are remotely homologous to each other, which makes it difficult for RNAalifold to calculate common secondary structures from alignments produced by CLUSTAL-W. INFERNAL searches the common secondary structure of the query sequences for a given sequence, and thus the CM search fails if no acceptable covariance model for the query sequences can be generated. Although using structural alignments for ncRNAs might improve the homology search with INFERNAL, it is not certain that given query sequences have common secondary structures. In such cases, it is difficult for any alignment programs to produce robust alignments with acceptable common secondary structures. In fact, the secondary structures of snoRNAs used in our experiments are highly diverse so that we often did not obtain suitable multiple alignments for building CMs even if using structural alignment programs (data not shown). In contrast, SVMs calculate kernel values, i.e., pairwise similarities, between every pair of examples, and learn the weight parameters for each example in order to maximize the margin between the positives and the negatives. After this, the trained SVMs predict the classification of a new example based on the weighted sum of kernel values of the new example and all the training examples. In other words, SVMs make a decision about the classification based on the majority voting principle with respect to the optimized weights. This approach minimizes the risk of mispredictions and makes decisions which are more robust than those of the methods which use only one representative such as a common secondary structure of the query sequences, that is, SVMs with our kernel require no common secondary structures of the query sequences, and can make robust predictions in performing remote homology search of structural ncRNAs. This approach, however, requires a number of kernel computations for each sequence to be analyzed, proportional to the number of support vectors collected in training SVMs. Therefore, the prediction process should take a long computation time if the training process could not reduce the number of support vectors.

### Kernel hierarchical clustering

**Figure 5:**
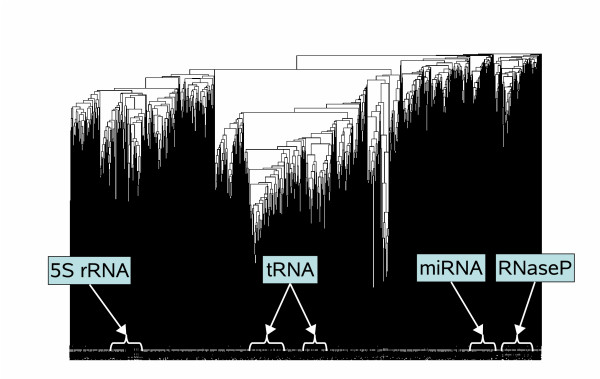
**The dendrogram resulting from applying our kernel and WPGMA to the dataset**. Some clusters containing typical families are indicated, such as 5S rRNA, tRNA, miRNA and RNaseP. This dendrogram was produced from Additional file 1 which is a newick format file calculated by our kernel and WPGMA. A magnifiable version of this dendrogram is available as Additional file 2.

**Figure 6:**
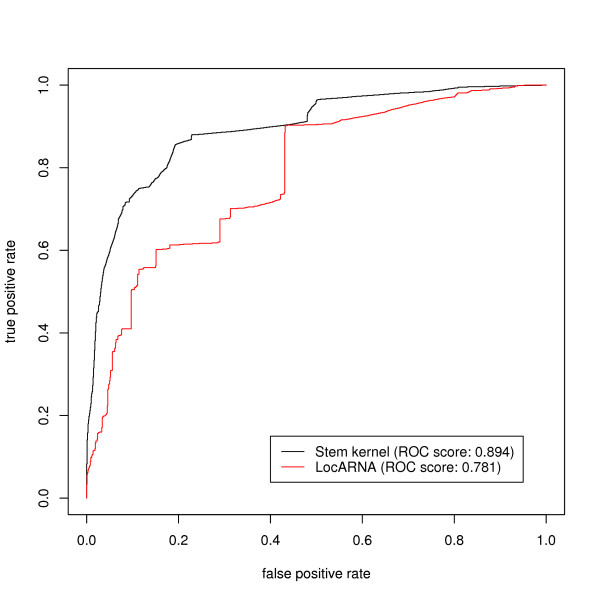
ROC curves of the degree of agreement between the clustering and the Rfam families in comparing our kernels with LocARNA.

We attempted to attain a kernel hierarchical clustering using the weighted pair group method algorithm (WPGMA) with the stem kernels for the same dataset as [36], extracted from the Rfam database [26], which contains 503 ncRNA families and a total of 3,901 sequences that have no more than 80% sequence identity and do not exceed 400 nt in length. Figure 5 shows the resulting dendrogram of the dataset, indicating some typical families, where sequences of the same family are likely to be contained in the same cluster (see also Additional files 1 &2. We evaluated the degree of agreement between the obtained clusters and the Rfam classification by converting the problem of cluster comparison into a binary classification problem in the same way as described in [36]: For every clustering cutoff threshold of the distance on the dendrogram, let the number of true positives (*TP*) be the number of sequence pairs in the same cluster which belong to the same family of Rfam. Analogously, let the number of false positives (*FP*) be the number of sequence pairs in the same cluster which belong to different families, the number of false negatives (*FN*) be the number of sequence pairs from the same family which lie in different clusters, and the number of true negatives (*TN*) be the number of sequence pairs from different families which lie in different clusters. The ROC curve plots the true positive rates as a function of the false positive rates for different clustering thresholds. Figure 6 shows the ROC curves for our kernel and LocARNA [36]. LocARNA produced hierarchical clusters whose ROC score was 0.781, while our kernel produced a score of 0.894.

LocARNA and the DAG-based stem kernels are similar to each other in their approximation technique, in which the base pairs whose base-pairing probability is below a predefined threshold are disregarded. One of the most important differences between the above two methods is that LocARNA calculates a score for only the best scoring secondary structure with bifurcations, while stem kernels sum all scores over an ensemble of common stem structures, including any suboptimal structures. In other words, stem kernels can be regarded as a variant of Sankoff algorithm [37], which calculates the partition function without any bifurcations. This feature of stem kernels determines their robustness with respect to measuring structural similarities.

## Conclusion

We have developed a new technique for analyzing structural RNA sequences using kernel methods. This technique is based on directed acyclic graphs (DAGs) derived from base-pairing probability matrices of RNA sequences, and significantly reduces the computation time for stem kernels. Our method considers only likely base pairs whose base-pairing probability is above a predefined threshold. The kernel values are calculated using DAG kernels, where each DAG is produced from these likely base pairs. Furthermore, we have proposed profile-profile stem kernels for multiple alignments of RNA sequences, which utilize the averaged base-pairing probability matrices of multiple alignments of RNA sequences.

Our kernels outperformed the existing methods for detection of known ncRNAs by using SVMs and kernel hierarchical clustering. In the experiments where SVMs were used, the stem kernels performed nearly perfect discrimination in the dataset, and collected a smaller number of support vectors in comparison with the local alignment kernels due to the robustness of the stem kernels with respect to secondary structures. Therefore, stem kernels can be used for reliable similarity measurements of structural RNAs, and can be utilized in various applications using kernel methods.

The new technique proposed in this paper significantly increases the computation speed for stem kernels, which is a step toward the realization of a genome-scale search of ncRNAs using stem kernels. Since our method is capable of detecting remote homology, it is possible to discover new ncRNAs which cannot be detected with existing methods.

## Availability

Our implementation of the profile-profile stem kernels is available at  under the GNU public license. It takes RNA sequences or multiple alignments, and calculates a kernel matrix, which can be used as an input for a popular SVM tool called LIBSVM [38]. Furthermore, our software is capable of parallel processing using the Message Passing Interface (MPI) [39].

## Authors' contributions

KS developed the algorithm, wrote the code and performed all experiments. TM, KA and YS provided helpful insights in the experiments and the discussion, while YS initiated the project. KS drafted the manuscript. All authors read and approved the final manuscript.

## Supplementary Material

Additional file 1 **A newick format file used for drawing Figure **5. Figure 5 was produced from this file using the R ape package . Click here for file

Additional file 2 **A magnifiable version of Figure **5. Similarly to Figure 5, this figure was produced from Additional file 1 using the R ape package, and was stored in PDF format in order to enable magnification. Click here for file
