# Supplementary material for: Directed acyclic graph kernels for structural RNA analysis
Source: BMC Bioinformatics. 2008 Jul 22;9:318. doi: 10.1186/1471-2105-9-318 (PMC2515856; doi:10.1186/1471-2105-9-318)

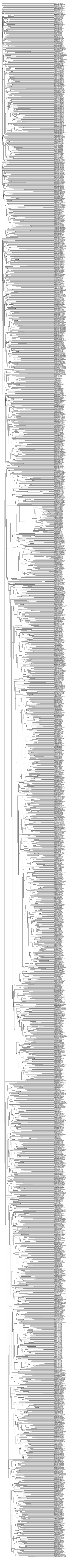

Supplement: Additional file 2 — A magnifiable version of Figure 5. Similarly to Figure 5, this figure was produced from Additional file 1 using the R ape package, and was stored in PDF format in order to enable magnification. [file 1471-2105-9-318-S2.pdf]
